# Bacteriophage P1*vir*-induced cell-to-cell plasmid transformation in *Escherichia coli*

**DOI:** 10.3934/microbiol.2017.4.784

**Published:** 2017-10-10

**Authors:** Chiaki Sugiura, Saki Miyaue, Yuka Shibata, Akiko Matsumoto, Sumio Maeda

**Affiliations:** Graduate School of Humanities and Sciences, Nara Women's University, Kitauoya-nishimachi, Nara 630-8506, Japan

**Keywords:** horizontal gene transfer, *Escherichia coli*, transformation, P1 phage, transduction

## Abstract

Bacteria undergo horizontal gene transfer via various mechanisms. We recently reported that cell-to-cell transfer of nonconjugative plasmids occurs between strains of *Escherichia coli* in co-cultures, and that a specific strain (CAG18439) causes frequent plasmid transfer involving a DNase-sensitive mechanism, which we termed “cell-to-cell transformation”. Here we found that CAG18439 is a type of P1 bacteriophage lysogen that continuously releases phages. We tested the ability of P1*vir* bacteriophage to induce horizontal plasmid transfer and demonstrated that such a horizontal plasmid transfer was caused by adding culture supernatants of P1*vir*-infected cells harboring plasmids to other plasmid-free cells. This plasmid transfer system also reproduced the major features of plasmid transfer involving CAG18439, suggesting that P1*vir*-induced plasmid transfer is equivalent or very similar to plasmid transfer involving CAG18439. We further revealed that approximately two-thirds of the P1*vir*-induced plasmid transfer was DNase-sensitive, but that complete abolition of plasmid transfer was observed when proteins were denatured or removed, despite the presence or absence of DNase. Therefore, we concluded that P1*vir*-induced plasmid transfer is largely due to the occurrence of cell-to-cell transformation, which involves the assistance of some proteinaceous factor, and partly due to the occurrence of plasmid transduction, which is mediated by phage virions. This is the first demonstration of the P1-phage-induced cell-to-cell transformation.

## 1. Introduction

Horizontal gene transfer is an important mechanism through which bacteria adapt to various environmental changes and evolve for better survival [1],[2]. Although three gene-transfer mechanisms—transformation, conjugation, and transduction—are well-known [1], several variations of these mechanisms have recently been suggested [3],[4],[5]. Bacteriophages generally mediate horizontal gene transfer by transduction [1],[6]. Plasmid transduction, which transfers intracellular plasmids from donor to recipient cells, has been reported in several phages [7],[8], although not many examples have been reported so far. Phage P1 is popular because of its ability to induce generalized transduction in *Escherichia coli* [6], and several reports have described plasmid transduction by P1 [9],[10]. In addition to transduction, transformation of DNA, released from phage-infected lysed cells, by neighboring cells is also generally considered to be feasible [1],[11]; however, no experimental evidence of this type of transformation in *E. coli* has been presented.

Over the last several years, we have revealed that a low frequency of spontaneous lateral transfer of non-conjugative plasmids occurs in an *E. coli* mixed cell culture in a colony biofilm (a biofilm formed on an air-solid surface [12]) [13],[14]. We later found that specific combinations, including that of the strain CAG18439 and the plasmid pHSG299, show highly frequent lateral plasmid transfer even in liquid culture [15]. By using this experimental set-up, we have demonstrated the involvement of a DNase-sensitive transfer mechanism [15], a proteinaceous extracellular promoting factor derived from CAG18439 [15], and a specific promoting DNA sequence of 88-bp on pHSG299 [16]. We termed this transformation “cell-to-cell transformation” [15]. Subsequent studies of genome-wide screens for the genes involved in DNA acquisition in recipient cells have suggested that this plasmid transfer does not involve simple transformation but is instead a complicated phenomenon [17],[18],[19].

In this study, we discovered that strain CAG18439 is a kind of P1 lysogen, probably that of P1*vir* or its derivative. Therefore, we examined the abilities of P1*vir* to induce cell-to-cell plasmid transfer, and we demonstrated that plasmid transfer could be caused by adding the culture supernatants of P1*vir*-infected strains harboring plasmids to other, plasmid-free, strains. Here, we present data on the features of this phenomenon, discuss its possible mechanisms and similarity to plasmid transfer involving CAG18439, and propose that P1*vir*-induced horizontal plasmid transfer occurs largely by cell-to-cell transformation and partly by plasmid transduction.

## 2. Materials and Method

### 2.1. *E. coli* strains, plasmids, and materials

The following *E. coli* strains, plasmids, and P1*vir* phage DNA (HR16) were obtained from the National BioResource Project (NBRP), *E. coli* (http://www.shigen.nig.ac.jp/ecoli/strain/top/top.jsp): DH5 [20], MG1655 [21], HB101 [22], MC4100 [23], CAG18439 [24], BW25113 [25], Keio collection strains [25], pHSG299 [26], and pHSG399 [26]. Plasmids pHSG399F6, pHSG299ΔF6, and pHSG299cam were constructed as previously described [15],[16]. Details of abovementioned *E. coli* strains and plasmids are shown in Table 1. Tetracycline, streptomycin, chloramphenicol, and Luria-Bertani (LB) powder (Lennox) were obtained from Sigma. Tryptic soy broth (TSB) was supplied by Becton, Dickinson. Distilled water (DNase- and RNase-free, molecular biology grade) and kanamycin (kan) were obtained from Invitrogen. DNase I (bovine pancreas, Grade II) was supplied by Boehringer Mannheim. Syringe filters for sterilization (pore size: 0.20 µm) were obtained from Iwaki. Agar powder (guaranteed-reagent grade), proteinase K, trypsin, and other general reagents were obtained from Wako.

**Table 1.** *E. coli* strains and plasmids used in this study.

Strain, phage, or plasmid	Genotype or characteristics	Reference or source
**Strains**		
DH5	F^−^, *deoR, recA1, endA1, hsdR17(rK^−^, mK^+^), supE44, λ^−^, thi-1, gyrA96, relA1*	[20]
MG1655	F^−^, *λ^−^, rph-1*	[21]
HB101	F^−^, *λ^−^, hsdS20(r^−^_B_, m^−^_B_), recA13, ara-14, proA2, lacY1, galK2, rpsL20(str^r^), xyl-5, mtl-1, supE44, leu, thi*	[22]
MC4100	F^−^, *araD139,* Δ*(lacZYA-argF)U169, deoC1, flbB5301, ptsF25, relA1, rbsR, rpsL150(str^r^)*	[23]
CAG18439	MG1655 derivative; F^−^, *λ^−^, lacZ118(Oc), lacI3042::Tn10(tet^r^), rph-1*	[24]
BW25113	The strain of origin of the Keio Collection; F^−^, *rrnB,* Δ*lacZ4787, HsdR514,* Δ*(araBAD)567,* Δ*(rhaBAD)568, rph-1*	[25]
Keio strains(Δ*yfgA,* Δ*ycjU* Δ*ygcO,* Δ*ybcS,* Δ*yihO,* Δ*ymfL*)	BW25113 derivatives, Δ*(single gene)::kan^r^*	[25]

**Phage**		
P1*vir*	HR16	Obtained from NBRP

**Plasmids**		
pHSG299	*kan^r^*; a pUC-like high-copy cloning vector containing the pMB1 origin	[26]
pHSG399	*cam^r^*; a pUC-like high-copy cloning vector similar to pHSG299	[26]
pHSG299cam	*cam^r^*; pHSG299 containing the *cam^r^* gene of pHSG399	[15]
pHSG299ΔF6	pHSG299 lacking CTPS	[16]
pHSG399F6	pHSG399 containing CTPS	[16]

### 2.2. Preparation of seed suspension of P1*vir* and P1*vir*-derived lysogens

All the following manipulations on P1*vir* were performed in accordance with conventional methods as described [20],[27],[28]. P1*vir* phage particles were reformed by transformation of the DNA obtained from NBRP to MG1655 and amplified in liquid or soft agar medium. After purification of these phage particles from the culture supernatant by centrifugation, DNase treatment to remove *E. coli* DNA, and filtration to remove cell residue, they were stored at 4 °C as the seed stocks for experiments. P1*vir* infection of MG1655 or other *E. coli* K-12 strains also produced some lysogenic cells that are resistant to P1*vir* and can constantly produce phage particles under non-inducing conditions. In the following text, we call the lysogen produced by P1*vir* and phage produced by the lysogen “P1*vir*-derived lysogen” and “P1*vir*-derived phage” respectively.

### 2.3. Phage DNA isolation and analysis

Phage DNAs were isolated from phage particles (purified by the method described in section 2.2) from plate lysate or culture supernatant of P1*vir*-infected cells or strain CAG18439 using conventional methods [28], digested with BamHI, EcoRI, or KpnI, and analyzed by 0.8% (w/v) agarose-tris-borate-EDTA (TBE) gel electrophoresis [28].

### 2.4. Plasmid transfer experiment with culture supernatant of P1*vir*-infected cells or P1*vir*-derived lysogens harboring plasmids

Horizontal plasmid transfer experiments with culture supernatant of P1*vir*-infected cells were performed. Briefly, plasmid-harboring cells (donor cells) were precultured alone in LB broth containing an appropriate antibiotic, then cultured in fresh antibiotic-free LB broth (5 mL) with seed suspension (25 µL) of P1*vir* at 37 °C for 16 h with shaking (225 rpm). A small amount (1/200 dilution) of the carry-over of an antibiotic contained in the preculture media did not influence the cell growth in the following steps. The supernatant of this culture was prepared by centrifugation and subsequent filtration with a membrane filter (pore size: 0.20 µm). This culture supernatant (10 µL) and the precultured plasmid-free cells (recipient cells: 4 × 10^7^ cells) were added to fresh TSB (1 mL) in each well of a 96-deep-well microplate and cultured at 37 °C for 16 h with shaking (600 rpm). After centrifugation of this culture, the cells were recovered, diluted with LB broth, and spread onto LB agar plates containing suitable antibiotics to detect transformants. After overnight incubation at 37 °C, the colonies produced were counted. Some of these colonies were used for plasmid isolation and analysis. The frequency of plasmid transfer was calculated as the ratio of the number of transformants to the number of recipient cells, which was deduced from the OD_600_ value of the cell suspension just before plating.

Similar experiments using plasmid-harboring P1*vir*-derived lysogens as plasmid donors were performed by using the same protocol as mentioned above, except that the culture supernatant for horizontal plasmid transfer was directly prepared from the culture of plasmid-harboring P1*vir*-derived lysogens.

### 2.5. Treatment of culture supernatant of P1*vir*-infected cells with DNase I, heat, or phenol-chloroform extraction and ethanol precipitation

Culture supernatants of P1*vir*-infected cells were treated with DNase I, heat, or phenol-chloroform extraction and ethanol precipitation as described below. For DNase I treatment, fresh TSB (1 mL) and DNase I (300 µg) were added to 10 µL of the prepared culture supernatant of P1*vir*-infected cells, and this solution was incubated at 37 °C for 1 h. Then recipient cells (4 × 10^7^ cells) were added to this solution to start a plasmid transfer experiment. For heat treatment, the prepared culture supernatant of P1*vir*-infected cells was heated at 98 °C for 15 min, and then cooled to 37 °C. For phenol-chloroform and ethanol precipitation treatment, the prepared culture supernatant of P1*vir*-infected cells was treated with phenol-chloroform, and subjected to ethanol precipitation by using the conventional method [28]. This supernatant was then dissolved in PBS.

## 3. Results

### 3.1. Strain CAG18439 is a P1 lysogen

We previously showed that the *E.coli* strain CAG18439 as both plasmid donor and recipient caused frequent plasmid transfer in a mixed cell culture [15]. CAG18439 (Table 2) was originally established by Singer et al. [24] by means of P1 transduction of MG1655 cells with P1*vir* [29]. In our experiments using CAG18439, we noticed that the culture supernatant of CAG18439 had plaque-forming activity (Table 2). This activity has been recently confirmed in the original stocks of CAG18439 and other multiple CAG-series strains in the deposit organization from which we had obtained CAG18439 (see https://shigen.nig.ac.jp/ecoli/strain/resource/strainGeneMutant/detail/226). Therefore, it is believed that plaque-forming lysogenic phages are commonly present in some parts of the original CAG-series strains. This suggests that P1*vir* or its derivative is the most probable candidate for the lysogenic phage commonly present in the CAG-series strains. It should be noted that supernatants of all other strains used in this study (listed in Table 1) showed no plaque-forming activity.

**Table 2.** Plaque assay using culture supernatants of CAG18439 and P1*vir*-infected MG1655.

Concentration of culture supernatant (%)	Titer (pfu/mL)	
	P1*vir*	CAG18439
1	CL	CL
10^−1^	CL	215
10^−2^	CL	28
10^−3^	155	3.4

CL, confluent lysis.

**Figure 1.**
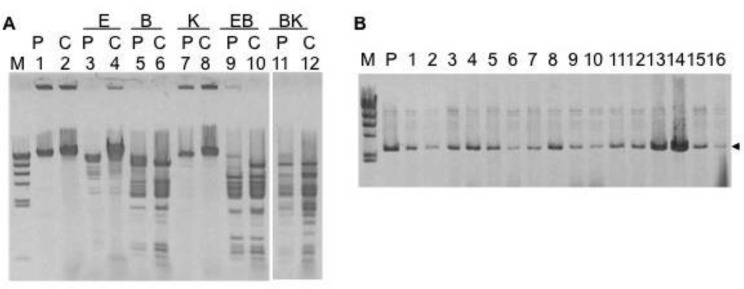
A: Restriction enzyme analysis of DNA of P1*vir* phage and DNA of phage produced from CAG18439. Phage DNAs were isolated as described in the Materials and Method. The DNAs were digested by restriction enzymes and subjected to 0.8% (w/v) agarose-TBE gel electrophoresis. Odd-numbered lanes (shown by “P”) represent DNA of P1*vir* phage, and even-numbered lanes (shown by “C”) represent that of CAG18439-derived phage. Lane M: size marker (Lambda Hind III); lanes 1 and 2: undigested DNAs; lanes 3 and 4: EcoRI-digested DNAs; lanes 5 and 6: BamHI-digested DNAs; lanes 7 and 8: KpnI-digested DNAs; lanes 9 and 10: DNAs digested by EcoRI and BamHI; lanes 11 and 12: DNAs digested by BamHI and KpnI. B: Plasmid analysis of colonies obtained in a P1*vir*-induced plasmid transfer experiment using MG1655 and MC4100 as recipients. Agarose gel electrophoresis of EcoRI-digested plasmids isolated from kan-resistant colonies. Arrowhead indicates the band for pHSG299 (2676 bp). Lane M: size marker (Lambda Hind III); lane P: positive control (pHSG299); lanes 1 to 16: plasmids obtained from the kan-resistant transformants produced. Lanes 1 to 8 represent plasmids of MG1655 as recipient, and lanes 9 to 16 represent those of MC4100. Plasmid donors are MG1655 (lanes 1, 2, 9, and 10), MC4100 (lanes 3, 4, 11, and 12), HB101 (lanes 5, 6, 13, and 14), and DH5 (lanes 7, 8, 15, and 16).

To identify the phage of CAG18439 further, we isolated DNA from phage particles in the DNase-treated culture supernatant of CAG18439 and performed a comparison with the DNA of P1*vir*. Restriction enzyme analysis of those DNAs revealed that the cutting patterns of the two DNAs were almost identical (Figure 1A). This indicated that the phage produced by CAG18439 was a kind of P1 phage; namely, CAG18439 was a P1 lysogen, possibly that of P1*vir* itself or its spontaneous lysogenic revertant. In the following text, we call the lysogen produced by P1*vir* and the phage produced by the lysogen “P1*vir*-derived lysogen” and “P1*vir*-derived phage” respectively.

### 3.2. P1*vir* induces horizontal plasmid transfer via culture supernatant

The above result led us to hypothesize that the promoted horizontal plasmid transfer specific to CAG18439 may have been caused by the P1*vir*-derived phage lysogenized within cells of this strain and released from them into culture medium. To test this hypothesis, we examined the ability of P1*vir* to induce horizontal plasmid transfer (Table 3). MG1655 and several other strains harboring pHSG299 were infected with P1*vir*, and their culture supernatants were prepared by centrifugation and filtration to remove the cells completely. Each supernatant was added to TSB together with seed cells of MG1655 or MC4100 (as plasmid recipients) and cultured. The cultured cells were plated onto LB agar containing kan to detect cells that had acquired pHSG299. All of the culture supernatants of P1*vir*-infected cells produced kan-resistant colonies in both MG1655 and MC4100 as recipients (Table 3), whereas none of the culture supernatants of uninfected cells produced resistant colonies. A plasmid isolation experiment confirmed that the produced kan-resistant cells contained full-length pHSG299 (Figure 1B). Because this manner of plasmid transfer mediated by culture supernatant was the same as with CAG18439, as reported previously [15], we concluded that the above hypothesis was true. Notably, plasmid transfer also occurred when we used culture supernatants of P1*vir* lysogens harboring pHSG299 (P1*vir* lysogens that were transformed by pHSG299), instead of freshly infected cells as described above. This corresponds to the protocol in which CAG18439 harboring plasmids was used as a plasmid donor in our previous study [15]. In the following text, we call the plasmid transfer using a culture supernatant of P1*vir*-infected cells harboring plasmids (shown in Table 3) “P1*vir*-induced horizontal plasmid transfer” or “P1*vir*-induced plasmid transfer”.

### 3.3. Effect of the promoting sequence on P1*vir*-induced horizontal plasmid transfer

**Figure 2.**
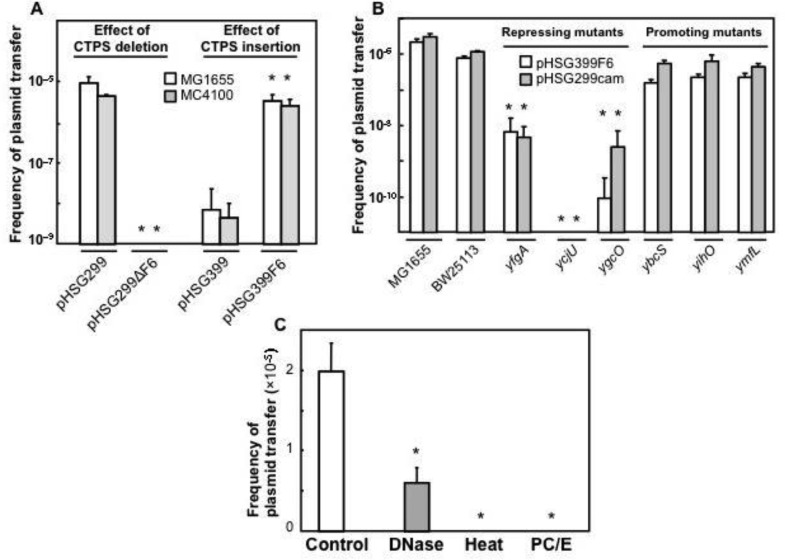
A: Effects of CTPS on plasmid transfer. Culture supernatant derived from P1*vir*-infected plasmid-donor cells (DH5), which harbor plasmids with (pHSG299 and pHSG399F6) or without (pHSG299ΔF6 and pHSG399) CTPS, was added to recipient MG1655 (white bar) or MC4100 (gray bar) in TSB. These mixtures were cultured and plated to assess the plasmid transfer, as described in the Materials and Method. Data on plasmid transfer frequency are presented as means and SD [* *t*-test: *P* < 0.05, *n* = 5; compared with each control (pHSG299 or pHSG399)]. B: Effects of single-gene knockout on plasmid transfer. Culture supernatant derived from P1*vir*-infected plasmid-donor cells (white bars: DH5 harboring pHSG399F6; gray bars: DH5 harboring pHSG299cam) was added to each recipient strain [MG1655, BW25113 (the strain of origin of Keio mutants), and Keio mutants (*yfgA*, *ycjU*, *ygcO*, *ybcS*, *yihO*, and *ymfL*)] and cultured. Data on plasmid transfer frequency are presented as means and SD (* *t*-test: *P* < 0.05, *n* = 5; compared with control BW25113). C: Effects of DNase I treatment, heating, or phenol-chloroform extraction and ethanol precipitation of culture supernatant derived from plasmid-donor cells on plasmid transfer. Culture supernatant derived from a P1*vir*-infected plasmid-donor strain (DH5 harboring pHSG299) was treated with DNase I (“DNase” bar), heating (“Heat” bar), or phenol-chloroform extraction and ethanol precipitation (“PC/E” bar), as described in the Materials and Method. The supernatant was then added to a recipient strain (MG1655), which was cultured. Data on plasmid transfer frequency are presented as means and SD (* *t*-test: *P* < 0.05, *n* = 5; compared with untreated control).

We previously showed that pHSG299, which shows frequent plasmid transfer in combination with CAG18439, contains an 88 bp promoting sequence (previously named CTPS [16], accession number: AB634455). To assess the promoting effect of the CTPS on P1*vir*-induced plasmid transfer, we examined several plasmids containing or not containing the CTPS (Figure 2A). Two plasmids containing CTPS (pHSG299 and pHSG399F6) had significantly higher transfer activities than those of the corresponding CTPS-free plasmids (pHSG299ΔF6 and pHSG399). This result indicated that CTPS also worked as a promoting sequence for P1*vir*-induced plasmid transfer.

**Table 3.** Horizontal plasmid transfer via culture supernatants of P1*vir*-infected strains harboring pHSG299.

Donor strain harboring pHSG299 infected with P1*vir*	Recipient strain	
	MG1655	MC4100
MG1655	+	+
MC4100	+	+
HB101	+	+
DH5	+	+

Frequency of plasmid transfer: +, 1E−6 to 1E−5.

### 3.4. Effect of single-gene knockout on P1*vir*-induced horizontal plasmid transfer

In previous experiments using CAG18439 cells harboring CTPS-containing plasmid as donors, we showed that specific single-gene knockout mutations in recipients could repress or promote plasmid transfer [17],[18]. Therefore, we tested the effects of these mutants on P1*vir*-induced plasmid transfer. We used three repressing mutants (Δ*yfgA* = *rodZ*, Δ*ycjU*, and Δ*ygcO*) [17] and three promoting mutants (Δ*ybcS*, Δ*yihO*, and Δ*ymfL*) [18] (Figure 2B). Use of these repressing mutants commonly resulted in a significant decrease in P1*vir*-induced plasmid transfer. This result was consistent with our previous result using CAG14839 [17], although the degree of repression in each mutant differed somewhat. In contrast, the use of the promoting mutants commonly resulted in neither an increase nor a decrease in P1*vir*-induced plasmid transfer. This result differed from our previous one using CAG14839 [18].

### 3.5. P1*vir*-induced horizontal plasmid transfer using P1*vir*-derived lysogens as recipients

We previously showed that, in some cases, CAG18439 works not only as a donor but also as a recipient in highly frequent plasmid transfer [15]. To test a similar set-up in P1*vir*-induced plasmid transfer, we used P1*vir*-derived lysogens as recipients (Table 4). P1*vir*-induced plasmid transfer also occurred in the lysogens as recipients, indicating that lysogenization with P1*vir*-derived phage does not abolish the ability to act as a recipient. This result was also consistent with our previous result using CAG14839 [15].

**Table 4.** P1*vir*-induced horizontal plasmid transfer using P1*vir* lysogens as recipients.

Donor strain harboring pHSG299 infected with P1*vir*	Recipient	Presence of P1*vir* in recipients	
		Non-lysogen	Lysogen
MG1655	MG1655	++	++
	MC4100	++	+
DH5	MG1655	++	++
	MC4100	++	+

Frequency of plasmid transfer: ++, 1E−6 to 1E−5; +, 1E−7 to 1E−6.

### 3.6. DNase sensitivity and functional protein dependence of P1*vir*-induced horizontal plasmid transfer

In the above experiments, we used filtered culture supernatants of P1*vir*-infected cells harboring plasmids as a plasmid-transfer source. Such supernatants should contain extracellular naked DNA released from lysed cells in addition to the phage virions. Indeed, we detected plasmid DNA by PCR analysis and active phage virions by plaque assay in the supernatants (data not shown). Therefore, both transformation and transduction are possible mechanisms of plasmid transfer in this system. DNase sensitivity is the key to distinguishing transformation and transduction, and in our previous study we demonstrated that cell-to-cell plasmid transfer with CAG18439 was largely DNase-sensitive [15]. To clarify the mechanism of P1*vir*-induced plasmid transfer and to confirm its similarity to plasmid transfer with CAG18439, we examined DNase sensitivity. DNase I treatment of a culture supernatant of P1*vir*-infected cells harboring plasmids significantly decreased the plasmid transfer frequency (Figure 2C), indicating that a DNase-sensitive mechanism is involved. However, this inhibitory effect was not complete (approximately one-third of plasmid-transfer activity remained after DNase I treatment; Figure 2C), implying the concurrent involvement of a DNase-resistant mechanism. Complete disappearance of plasmid transfer was observed when the supernatant was treated by simple heating (98 °C, 15 min) or phenol-chloroform extraction and ethanol precipitation (Figure 2C); such treatments should lead to the denaturing and removal of proteins but the retention of DNA. Therefore, this result suggested that some functional protein or proteins in the supernatant are needed for both the DNase-sensitive and the DNase-resistant transfer mechanisms. Together, these results suggested that P1*vir*-induced plasmid transfer comprised two different mechanisms: one that was dependent on DNase-accessible extracellular naked DNA and a protein or proteins in the supernatant, and another that was dependent on DNase-inaccessible DNA and some protein or proteins in the supernatant. Considering the probable factors in our experimental system, we concluded that the former mechanism involved a kind of transformation, and the latter involved transduction. The ratio of contributions of these two mechanisms, as estimated from the results shown in Figure 2C, was approximately 2 to 1 [transformation (DNase-sensitive part) to transduction (DNase-resistant part)].

## 4. Discussion

To investigate the mechanism of the frequent cell-to-cell plasmid transfer with CAG18439 that was previously reported [15]–[18], we examined the involvement of P1 phage. We obtained the following results: (1) CAG18439 is a P1 lysogen that continuously produces plaque-forming phage virions; (2) P1*vir* also induced horizontal plasmid transfer from plasmid-harboring cells to plasmid-free cells via the culture supernatant; (3) the previously reported promoting sequence (CTPS) [16] also promoted P1*vir*-induced plasmid transfer; (4) some of the previously reported knockout mutants [17],[18] also exhibited a similar effect on P1*vir*-induced plasmid transfer; (5) P1*vir*-derived lysogen also worked as a recipient in P1*vir*-induced plasmid transfer; and (6) P1*vir*-induced plasmid transfer was partly sensitive to DNase I and completely dependent on some functional protein or proteins in the culture supernatant. These results are consistent with most of the results of previous studies using CAG18439 [15],[16],[17] and can be explained by the hypothesis that P1*vir* phage (or P1*vir*-derived phage) infecting (or lysogenized within) plasmid-harboring cells can cause frequent horizontal plasmid transfer between *E. coli* cells via the culture medium. Therefore, plasmid transfer with CAG18439 is most probably induced by P1*vir* or P1*vir*-derived phage produced from CAG18439. Successful reproduction of essentially the same manner of plasmid transfer using P1*vir* without CAG18439 has clearly demonstrated the validity of this conclusion.

The reason for a discrepancy in the results of the promoting mutants (Figure 2B) is unknown at present. One possibility is that the difference of the experimental systems (co-culture in the previous study vs. separate culture in this study) resulted in this discrepancy. Alternatively, some differences between the phage of CAG18439 and the P1*vir* phage used in this study may have cause this discrepancy. In this respect, as well as for more clarification of the plasmid-transfer mechanism, further analysis of the phage of CAG18439 will be required.

The results in Figure 2C demonstrated that approximately two-thirds of the P1*vir*-induced plasmid transfer was executed by a DNase-sensitive mechanism. DNase sensitivity is a simple but reliable indicator to distinguish transformation from conjugation, transduction, and other DNase-resistant mechanisms [2],[30]. Therefore, we concluded that transformation is involved in two-thirds of this plasmid transfer. The data of Table 4, which indicates that plasmid-free lysogen can act as the recipient of this plasmid transfer, also support the conclusion that transformation, rather than transduction, preferentially occurs because lysogenic cells generally exhibit immunity (resistance to reinfection) against the same type of phages [1],[6]. This conclusion is also consistent with that of our previous study using CAG18439 [15]. It is logical to consider the transformation mechanism for P1*vir*-induced plasmid transfer because phage infection to plasmid-harboring cells (or spontaneous awakening of lysogenized phages in plasmid-harboring cells) naturally causes cell lysis and subsequent release of intracellular plasmid DNA usable for transformation. To our knowledge, this is the first demonstration of P1-phage-induced horizontal plasmid transfer by transformation.

However, it should be noted that P1*vir*-induced cell-to-cell plasmid transformation differs from the well-known simple transformation in *E. coli* [20] because this plasmid transfer is dependent on a specific DNA sequence (CTPS) in the transformed plasmid (Figure 2A) as well as on specific genes in the recipient cells (Figure 2B) and a functional protein or proteins released from P1-infectd cells into the culture medium (Figure 2C). This transformation is also characteristic in that its estimated frequency is rather high (∼10^−5^) (Figure 2C). In addition, we previously demonstrated that CAG18439 shows no promoting effect on simple natural transformation with purified plasmids [15]. These features indicate the novelty and uniqueness of this transformation mechanism, as suggested previously [15]–[19]. We speculate that a certain P1-phage protein or *E. coli* protein, whose size was estimated to be between 9 and 15 kDa [15], induced by P1 infection also helps with this transformation in an unknown manner that differs from that in transduction.

We suppose that a similar phage-induced transformation mechanism may work in cell-to-cell transformation between certain natural *E. coli* strains [30], although another phage-independent mechanism is also probably present because certain strain combinations consisting of exclusively lysogenic phage-free strains undergo cell-to-cell transformation [30],[31]. In addition, a low frequency of cell-to-cell transformation possibly occurs under certain conditions [13],[14]. We speculate that there are multiple mechanisms of cell-to-cell transformation and that they widely work in the environment.

We also found that part (approximately one-third) of the plasmid transfer was DNase resistant, suggesting the involvement of another DNase-resistant mechanism. Since treatments that simply denatured or removed proteins completely abolished P1*vir*-induced plasmid transfer (Figure 2C), we consider that the DNase-resistant, protein-dependent plasmid transfer via culture supernatant is due to plasmid transduction by P1*vir* or P1*vir*-derived phage, similar to the past examples [9],[10].

## 5. Conclusions

We revealed the occurrence of P1*vir*-induced cell-to-cell plasmid transformation in *E. coli* for the first time. This transformation occurs at a relatively high frequency via a special mechanism and differs from the well-known simple transformation in *E. coli*. This phenomenon may occur in the environment and is probably involved in the genetic dynamism and evolution of environmental *E. coli* and possibly other bacteria. Further investigations are required to obtain a complete understanding of the complex mechanisms behind this phenomenon.
